# Tactile information affects alternating visual percepts during binocular rivalry using naturalistic objects

**DOI:** 10.1186/s41235-022-00390-w

**Published:** 2022-05-11

**Authors:** Mikoto Ono, Nobuyuki Hirose, Shuji Mori

**Affiliations:** Department of Informatics, Graduate school of Information Science and Electrical Engineering, Kyushu University, 744 Motooka, Nishi-ku, Fukuoka City, Fukuoka 819-0395 Japan

## Abstract

**Introduction:**

Past studies have provided evidence that the effects of tactile stimulation on binocular rivalry are mediated by primitive features (orientation and spatial frequency) common in vision and touch. In this study, we examined whether such effects on binocular rivalry can be obtained through the roughness of naturalistic objects. In three experiments, the total dominant time of visual percepts of two objects was measured under binocular rivalry when participants touched one of the objects.

**Result:**

In Experiment 1, the total dominant time for the image of artificial turf and bathmat was prolonged by congruent tactile stimulation and shortened by incongruent tactile stimulation. In Experiment 2, we used the same stimuli but rotated their visual images in opposite directions. The dominant time for either image was prolonged by congruent tactile stimulation. In Experiment 3, we used different types of stimuli, smooth marble and rough fabric, and noted significant effects of the congruent and incongruent tactile stimulation on the dominant time of visual percepts.

**Conclusion:**

These three experiments demonstrated that visuo-tactile interaction on binocular rivalry can be mediated by roughness.

## Significance

This is the first study to demonstrate that binocular rivalry is mediated by tactile stimulation of roughness, which is one of the primary material properties. Visual and tactile information complement each other in human material perception. Past studied revealed visuo-tactile interaction of roughness in methods other than binocular rivalry. When binocular rivalry was used to investigate visuo-tactile interaction, the mediating features were spatial frequency and orientation. Our study adds new insights into the current understanding of material perception and visuo-tactile interaction.

In this study, visuo-tactile interaction was observed irrespective of semantic information of the stimuli used. Cross-modal interaction often takes place in semantic processes when the participants know the identities of stimuli. To eliminate such effects of semantic information, we presented two visual images rotated in opposite directions and had the participants report the dominant percepts of one of the rotated images. We confirmed visuo-tactile interaction in binocular rivalry.

## Introduction

Binocular rivalry occurs when competing images are dichoptically presented to each eye. One of the two images is dominantly perceived for a few seconds, after which the dominant image changes to the other and alternation takes place over time (Alais, 2012). Binocular rivalry is considered one of the useful tools to investigate the processes of underlying selective perception and visual awareness (Crick & Koch, 1998).

In the 1980s, it was suggested that binocular rivalry is due to interocular suppression between monocular neural representations of two images separately presented to the left and right eyes (e.g., Blake, 1989; Lehky, 1988; Varela & Singer, 1987; Wolfe, 1983; for a review, see Blake & Wilson, 2011). Interocular suppression is assumed to take place at earlier stages of visual processing (LGN and V1), and several studies demonstrated that LGN and V1 are involved in alternating visual percepts during binocular rivalry (e.g., Bartels & Logothetis, 2010; Haynes et al., 2005; Polonsky et al., 2000; Quinn & Arnold, 2010; Stuit et al., 2014).

However, the alternating visual percepts under binocular rivalry cannot be explained by only interocular suppression. For example, Logothetis et al. (1996) found that when two dichoptic stimuli were swapped between two eyes every 333 ms, perceptual alternation took place independently from the stimulus swapping. Kovács et al. (1996) reported that even when their participants were dichoptically presented with two patchwork stimuli composed of fragments of two coherent images, binocular rivalry occurred between intraocularly grouped coherent percepts. Furthermore, there is evidence from psychophysical (Wolf & Hochstein, 2011) and brain imaging studies (Lumer et al., 1998; Tong et al., 1998) that binocular rivalry is involved in higher visual areas and frontoparietal areas. The current view is that alternating visual percepts under binocular rivalry involve multiple areas of the brain (Alais & Blake, 2005; Blake & Logothetis, 2002; Blake & Wilson, 2011).

Several studies examined whether non-visual sensory information can influence alternating visual percepts during binocular rivalry. For example, Chen et al. (2011) examined the effects of auditory information. Their participants dichoptically viewed illustrations of a bird and a car while hearing sound and were asked to report the dominant image. Chen et al. compared the duration of perceiving the target image between under incongruent sound conditions (when the target image was a bird or a car, the sound was a car horn and engine-revving or a bird singing, respectively) and under irrelevant sound conditions (the sound of tableware clattering together in a restaurant). They found that the duration of dominant images was shortened by the presentation of incongruent sounds compared with irrelevant sound. They argued that the auditory information altered the visual percepts of object representation. Other studies also reported the effects of non-visual stimulation such as sound (e.g., Conrad et al., 2010, 2013; Pápai & Soto-Faraco, 2017; Plass et al., 2017), smell (Zhou et al., 2010, 2012) and hand motion (Maruya et al., 2007).

Tactile information also influences alternating visual percepts under binocular rivalry. van Ee et al. (2009) demonstrated that the duration of the dominant percept between two visual patterns (looming or rotation) under binocular rivalry is prolonged by vibrotactile stimulation synchronized with the rate of dynamic change in visual patterns. They argued that vibrotactile stimulus promotes attention to control over binocular rivalry.

Lunghi et al. (2010) conducted an experiment in which the participant dichoptically viewed two orthogonal grating stimuli and touched a grating tactile stimulus corresponding to the spatial frequency and orientation of one visual stimulus. The tactile stimulus congruent in orientation and spatial frequency with one of the visual gratings increased the probability of maintaining the dominant percept of that grating, and reduced the probability of switching to the other percept. They also conducted the experiment using visual and tactile grating stimuli of different spatial frequencies, and confirmed that the effects of tactile stimulation on the suppressed visual percept were selective for visuo-tactile spatial frequencies. Lunghi and Alais (2013), and Lunghi and Morrone (2013) reported that the visuo-tactile interaction on binocular rivalry is strictly selective for the orientation and spatial location of presentation stimuli. Furthermore, Lunghi and Alais (2015), and Lunghi et al. (2017) found that tactile stimulus congruent in orientation with the suppressed visual grating attenuated the suppression strength of visual stimulus during binocular rivalry and continuous flash suppression. Their studies suggested that vision and touch interact at early stages of visual processing prior to awareness, which has a narrow spatial frequency and orientation selectivity.

In the present study, we investigated whether the perception of roughness induces effects of tactile stimulation on binocular rivalry. Although spatial frequency and orientation are visual features that underlie many visual phenomena (Palmer, 1999), roughness is a primary tactile property that mediates surface and material perception of objects in touch (Okamoto et al., 2016) and has physiological correlates in the primary somatosensory cortex (Sathian et al., 2011; Servos et al., 2001; for a review, see Sathian, 2016). Several studies reported visuo-tactile interaction of roughness (for a review, see Klatzky & Lederman, 2010) and the ventral visual pathway exhibited neural activities corresponding to tactile roughness perception (Eck et al., 2016). It is thus possible that visuo-tactile interaction of roughness can alter binocular rivalry.

**Fig. 1:**
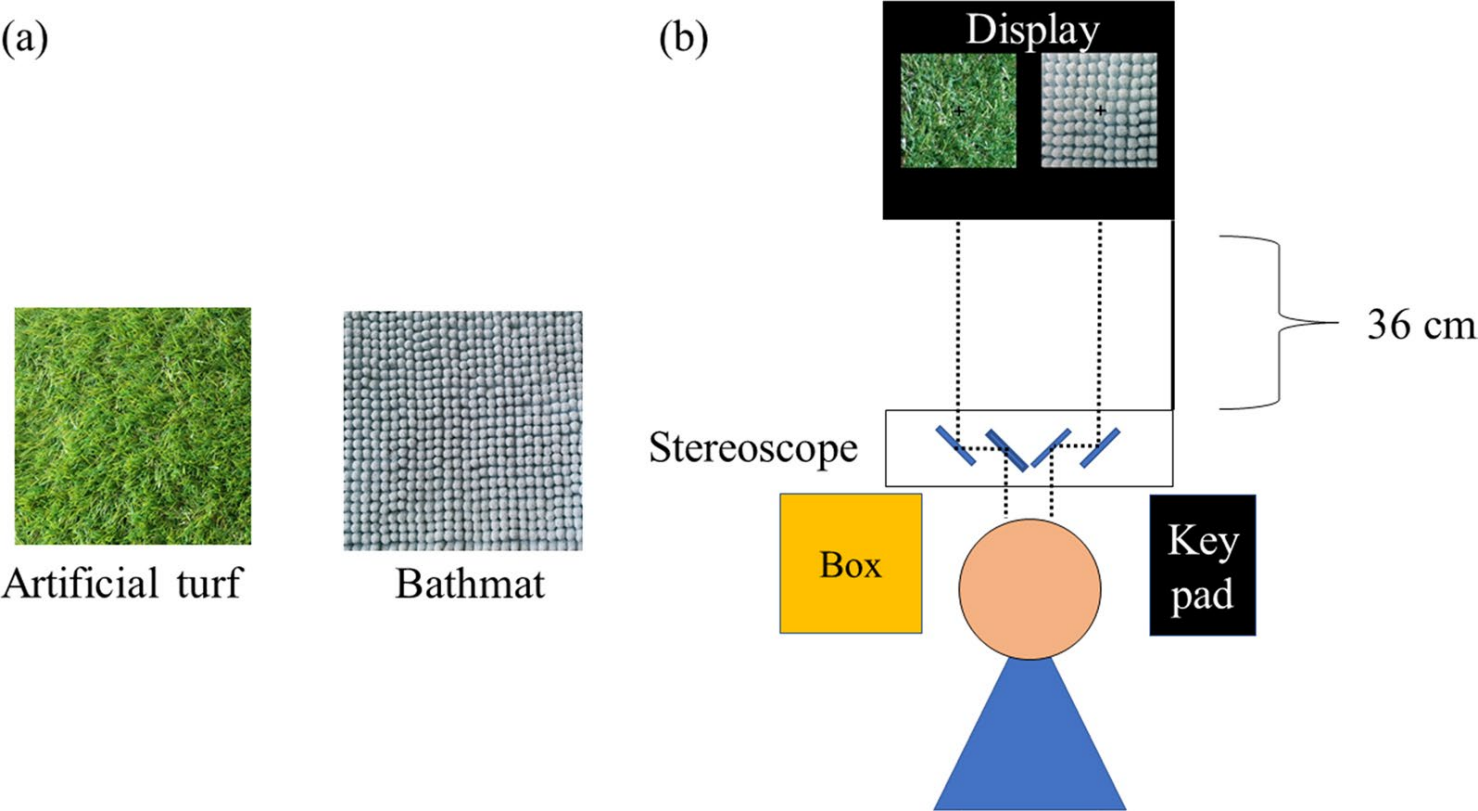
**a** Sample photo of tactile stimuli. **b** The environment in the experiment

As the stimuli, we used naturalistic objects, which were an artificial turf and a bathmat in Experiment 1 and 2 (Fig. 1), and marble and fabric in Experiment 3 (Fig. 6). In roughness studies, the majority have used sandpaper and dot patterns, which enable fine control of the degree of roughness on their surface (e.g., Connor et al., 1990; Eck et al., 2016; Kahrimanovic et al., 2009; Lederman, 1974, 1981; Meftah et al., 2000). We first employed these objects but were unable to induce binocular rivalry between their visual images presented to left and right eyes of the participants. The primary reason is that the images were not sufficiently different to compete for dominant percept, although their roughness was clearly different, visually and tactilely, from each other. We thus decided to use the naturalistic stimuli that induced binocular rivalry in our preliminary experiments, and had different degrees of tactile and visual roughness. To our knowledge, no study has used naturalistic stimuli to investigate visuo-tactile interaction in binocular rivalry, whereas naturalistic stimuli were found to be effective in inducing effects of auditory (Chen et al., 2011) and olfactory (Zhou et al., 2010) stimulation on binocular rivalry.

The present study consisted of three experiments. In Experiment 1, we used artificial turf and a bathmat, the former being perceived rougher than the latter, to investigate whether these stimuli will induce effects of tactile stimulation on binocular rivalry. In Experiment 2, we examined whether the effects of tactile stimulation observed with these stimuli are due to their semantic information. As the participants knew that the stimuli were artificial turf and a bathmat, they simply responded favorably when the visual and tactile stimuli were the same identity. To eliminate response bias due to semantic information, we had two visual stimuli rotated continuously, one in a clockwise direction and the other in a counter-clockwise direction, and asked the participants to report which of the rotations of images was dominant in perception. In Experiment 3, we used different types of stimuli, marble and fabric, to investigate whether the findings of the previous two experiments are generalizable to stimuli other than artificial turf and a bathmat.

## Experiment 1

### Methods

#### Participants

Twenty-two students from Kyushu University (13 males, 9 females, mean age 20.4 in a range of 18 to 24) participated for payment. They were unaware of the purpose of this experiment. Prior to the study, procedures were explained to the participants and their informed consent was received. All participants had normal or corrected-to-normal vision and all but one were right-handed.

#### Stimuli and apparatus

Tactile stimuli were patches of artificial turf and bathmat (approximately 25 × 25 cm in size, Fig. 1a). In order to be made invisible from the participant during the experiment, each stimulus was placed inside a box at the left front. The participant touched the surface of the stimulus with their left hand inside the box.

Visual stimuli were images of the two tactile stimuli, presented side-by-side on a CRT display with a black background (FMV-DP97X1, 0.04 cd/m^2^, 1024 × 768 resolution, 75-Hz refresh rate). The size of each image was 9.53° × 9.53° in visual angle, with a mean luminance of 36.17 and 40.50 cd/m^2^ for the artificial turf and bathmat, respectively. The size of our visual stimuli was rather large, and may have induced piecemeal rivalry in separate regions (Blake et al., 1992). Following the previous studies using naturalistic stimuli (Alais & Melcher, 2007), we chose this size because our stimuli had fine textures in the entire region that needed to be visible to the participants to elicit coherent rivalry of whole images. The presentation was controlled by Psychtoolbox-3 (Kleiner et al., 2007) in MATLAB. The participants observed the stimuli through a mirror stereoscope from a distance of approximately 36 cm (Fig. 1b) and a chin rest was used to minimize their head movements.

#### Procedure

**Fig. 2:**
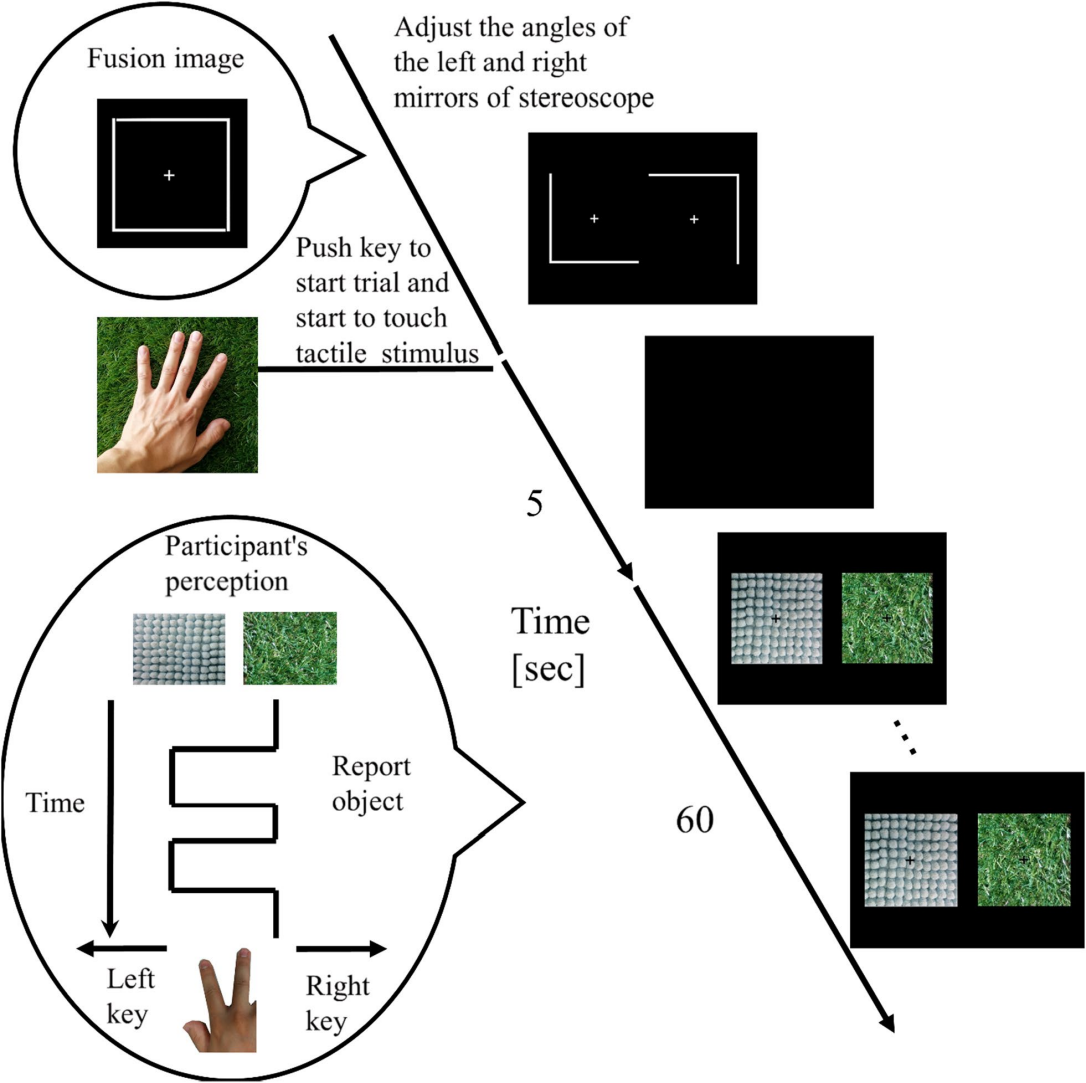
One trial sequence in the experiment

The experiment was conducted in a dark room. Participants wore ear plugs and headphones during the experiment to prevent hearing sound caused by touching the tactile stimuli. At the beginning of the experiment, each participant went through dark adaptation for three minutes and then adaptation to a white screen (96.4 cd/m^2^) on the CRT monitor for one minute. The flow of one trial is illustrated in Fig. 2. In each trial, the participant viewed the CRT monitor through the stereoscope, and was presented with a nonius line and a fixation cross (1.59° × 1.59°) to each eye. The distance from the fixation cross to the nonius line was 7.94°. The participant adjusted the angles of the left and right mirrors to make the two nonius lines fused into one square frame. The participant pressed a key when fusion was completed. Then, the display went blank for 5 s, during which they started to touch the tactile stimulus inside the box by moving their left hand clockwise. They were instructed to continue touching the tactile stimulus until the end of the trial. After 5 s, the participant was presented with two visual stimuli separately to left and right eyes for 60 s, during which they reported the dominant image by pressing one of two keys (left key: bathmat, right key: artificial turf) with their right hand. The participant was instructed to hold down a key corresponding to the dominant image that occupied more than 50% of the percept. We defined the dominant time for each image as the duration of corresponding key press.

There were three tactile stimulus conditions, i.e., bathmat, artificial turf, and no-stimulus. For the no-stimulus condition, no tactile stimulus was presented while the participant viewed the visual stimuli. The participant performed three blocks of two trials for each tactile stimulus condition, for a total of 18 trials. The locations of two visual stimuli were swapped between two trials in a block. The order of three tactile stimulus conditions and the locations of visual stimuli in the first trial in each block were randomized. The experiment lasted for approximately 1 h.

### Results

**Fig. 3:**
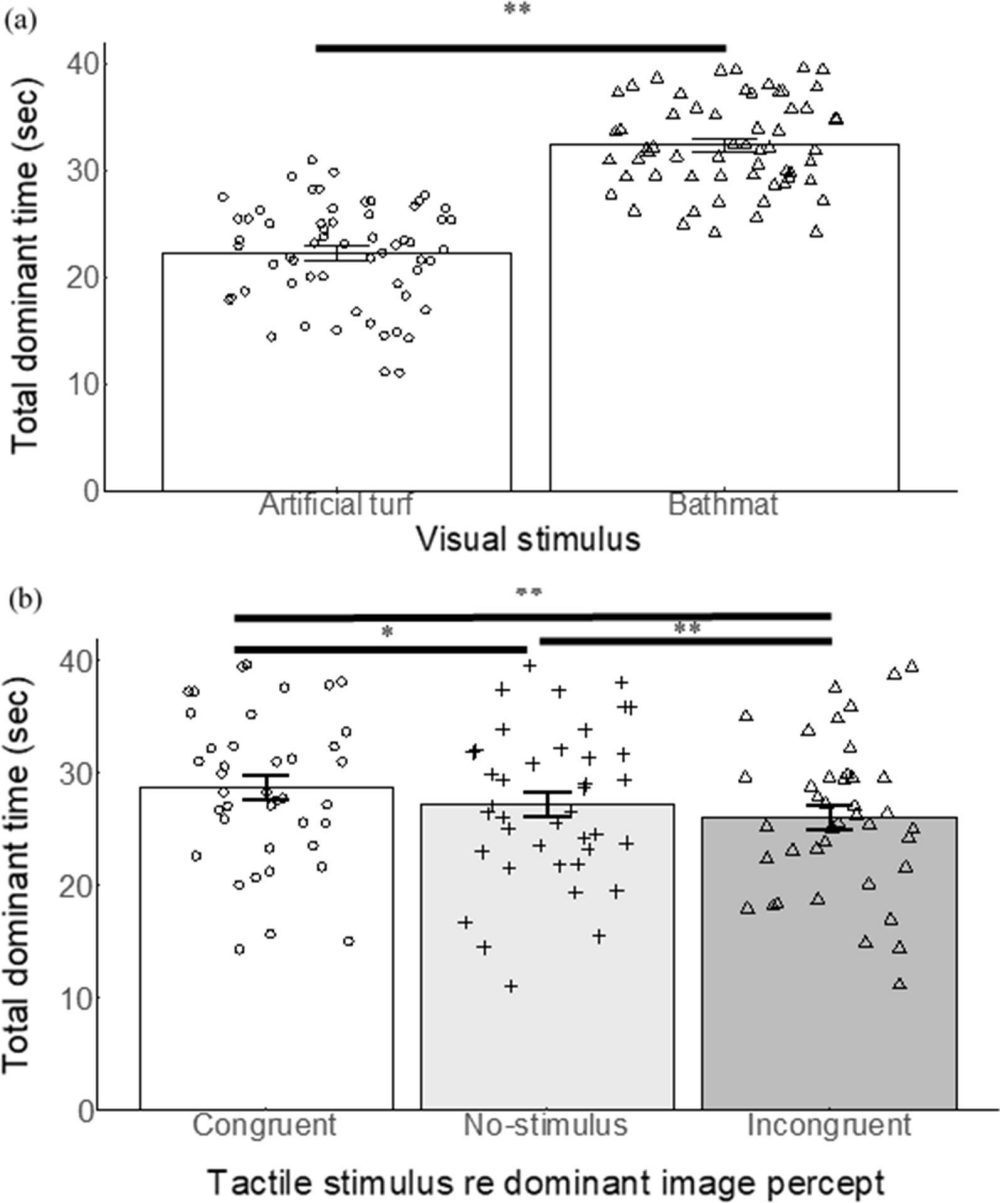
**a** The mean total dominant time for the images of artificial turf and bathmat. **b** The mean total dominant time for tactile stimulus regarding the dominant image. Error bars represent ± 1 SEM. Each dot represents individual data in each condition. * and ** refer to *p* < 0.05, and 0.01, respectively

Data from two participants were removed because they did not report alternating percepts during binocular viewing. For each participant, we computed the sum of the dominant time of the percept of each image in each trial and referred to it as the total dominant time. The mean total dominant times of twenty participants for the different visual images and for the tactile stimulus regarding the dominant image are shown in Fig. 3a, b, respectively. Note in Fig. 3a that the sum of the mean dominant time for the two images was not equal to 60 s because there was time when the participants did not push either key, moving their fingers from one key to the other. The two-way repeated-measures analysis of variance was performed on the total dominant time with two factors, dominant image (artificial turf or bathmat) and tactile stimulus regarding the dominant image (congruent, no-stimulus or incongruent). The main effect of the dominant image was significant (*F* (1, 19) = 30.834, *p* < 0.001, ${\eta }_{p}^{2}$= 0.619), indicating that the total dominant time was significantly longer for the bathmat than for the artificial turf. The main effect of tactile stimulus was also significant (*F* (2, 38) = 12.739, *p* < 0.001, ${\eta }_{p}^{2}$= 0.401). There was no significant interaction between the two factors (*F* (2, 38) = 0.015, *p* = 0.861, ${\eta }_{p}^{2}$ = 0.008). We conducted post-hoc tests for the main effect of tactile stimulus factor. For these tests, the overall *α* level of 0.05 was preserved by Bonferroni correction. The total dominant time was significantly longer under the congruent condition than under the no-stimulus (*p* < 0.05) or incongruent condition (*p* < 0.01). The total dominant time was significantly shorter under the incongruent condition than under the no-stimulus condition (*p* < 0.01).

### Discussion

We used artificial turf and a bathmat, the former being perceived rougher than the latter, and investigated whether these stimuli induce effects of tactile stimulation on binocular rivalry. The total dominant time of the visual percept of each visual stimulus was significantly prolonged by the congruent tactile stimulus and significantly shortened by the incongruent tactile stimulus compared with that under the no-stimulus condition. As shown in Fig. 3a, the total dominant time of the bathmat was significantly longer than that of the artificial turf. This may have been due to the difference in luminance between the two images (see Stimulus and Apparatus). Previous studies have reported that a brighter image is likely to win the initial competition and be perceived longer than a less bright image under binocular rivalry (e.g., Levelt, 1965). The important point, however, is that the effects of visuo-tactile interaction were obtained regardless of whether the dominant percept was of the artificial turf or of the bathmat.

In Experiment 2, to investigate whether this interaction was caused by response bias due to knowledge of the stimulus identity, we used the same stimuli, artificial turf and bathmat, but rotated the visual stimuli, one clockwise and the other counter-clockwise, and had the participants report which of the two rotating images they were seeing.

## Experiment 2

### Methods

#### Participants

Twenty-one students from Kyushu University (11 males, 10 females, mean age 19.6 in a range of 18 to 21) participated for payment. They did not participate in Experiment 1. They were unaware of the purpose of this experiment. Prior to the study, procedures were explained to the participants and their informed consent was received. All participants had normal or corrected-to-normal vision, and all but two were right-handed.

#### Stimuli and apparatus

**Fig. 4:**
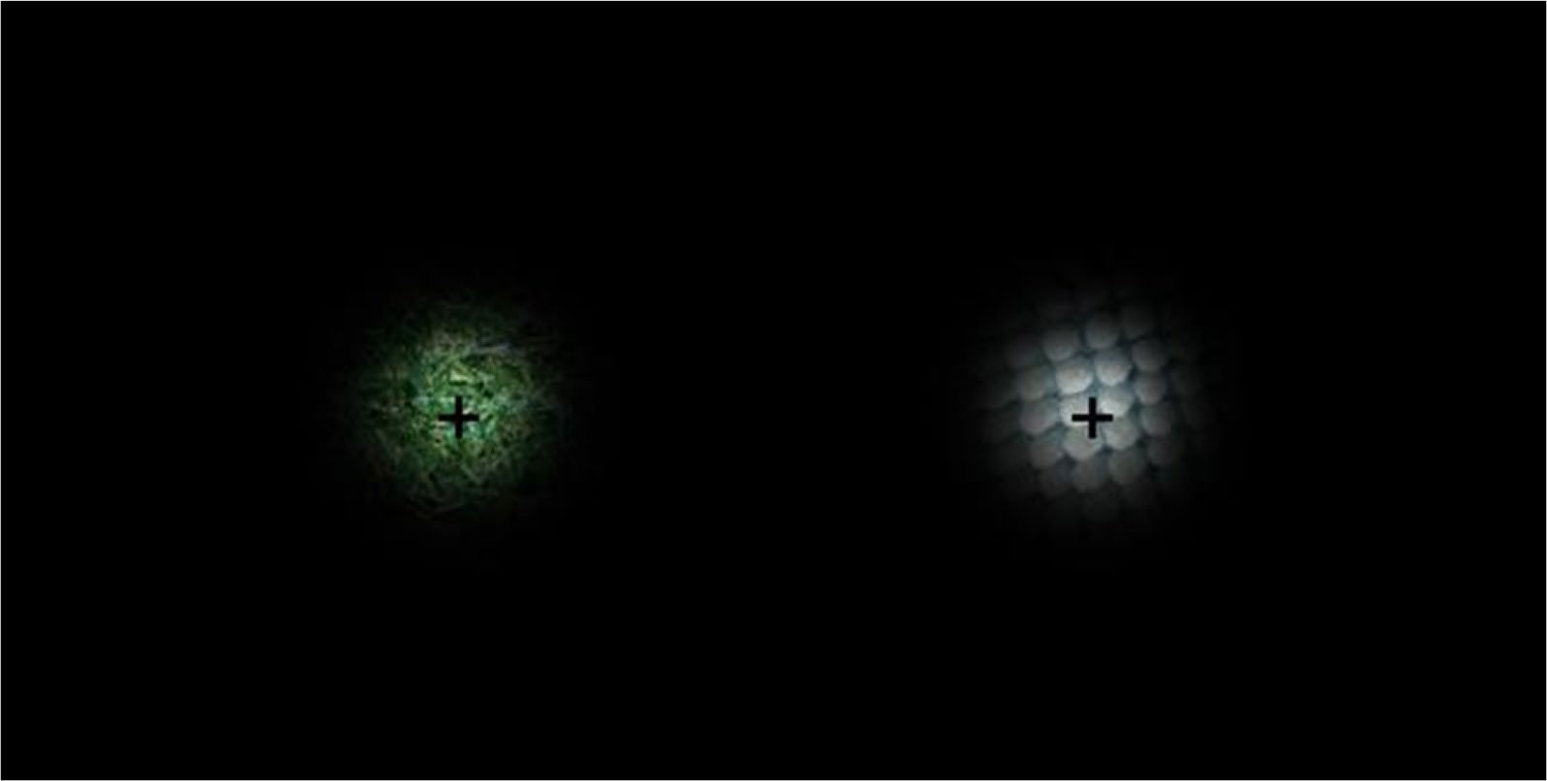
Image of presented visual stimuli on the CRT display

Visual stimuli were circularly cut-out images (6.4° in diameter) of those used in Experiment 1, with a mean luminance of 14.90 and 16.09 cd/m^2^ for the artificial turf and bathmat, respectively. In order to blur their edge, each image was windowed by 2D-Gaussian (*σ* = 20 pixel). The two stimuli were presented side-by-side on a CRT display with a black background (Fig. 4), and were rotated continuously during a 60 s observation period, one in a clockwise (CW) and the other in a counter-clockwise (CCW) direction, at a rate of one degree per frame (frame rate of 75 Hz). Tactile stimuli and apparatus were identical to those used in Experiment 1.

#### Procedure

The participant was presented with the two rotating visual stimuli separately to left and right eyes for 60 s, and were instructed to hold down by their right hand one of the two keys corresponding to the rotation directions (left key: CCW, right key: CW) of the dominant image that occupied more than 50% of the percept. We defined the dominant time for each image as the duration of corresponding key press.

As in Experiment 1, there were three tactile conditions (bathmat, artificial turf, and no-stimulus). For each tactile condition, the participant performed six blocks of two trials, for a total of 36 trials in this experiment. In each block, the rotation directions of the two visual stimuli were fixed, and their locations were swapped between the two trials. The same rotation direction for each visual stimulus was used in three blocks, although the stimulus locations in the first trial were randomized across the three blocks. The order of six blocks (three blocks for each rotation direction of the visual stimuli) was randomized in each tactile stimulus condition. The other procedures were identical to those in Experiment 1. The experiment lasted approximately 1 h.

### Results

**Fig. 5:**
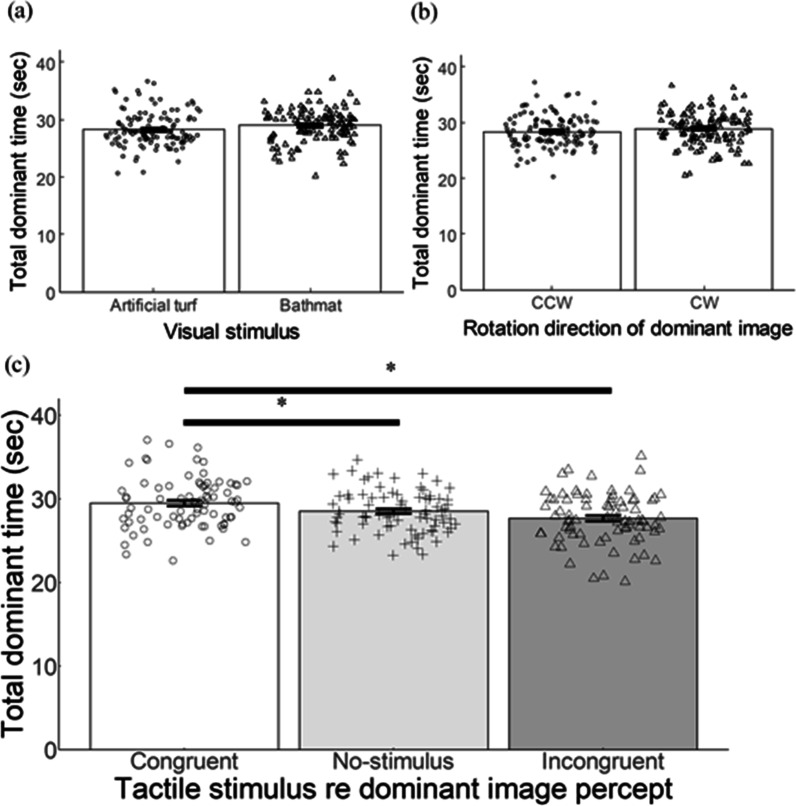
**a** The mean total dominant time for the images of artificial turf and bathmat. **b** The mean total dominant time for the rotation direction of dominant images for counter-clockwise (CCW) and clockwise (CW). **c** The mean total dominant time for tactile stimulus regarding the dominant image. Error bars represent ± 1 SEM. Each dot represents individual data in each condition. * refer to *p* < 0.05

Data from one participant were removed because they did not report alternating percepts during binocular viewing. For each of the other participants, we computed the sum of the dominant time of the percept of each image in each trial as the total dominant time. The mean total dominant times of twenty participants for the dominant images, for the rotation direction of dominant image, and for the tactile stimulus regarding the dominant image are shown in Fig. 5a–c, respectively. The three-way repeated-measures analysis of variance was performed on the total dominant time with three factors, dominant images (artificial turf or bathmat), tactile stimulus regarding the dominant image (congruent, no-stimulus, or incongruent), and rotation direction of dominant image (counter-clockwise: CCW or clockwise: CW). Neither the main effect of the dominant images (*F* (1, 19) = 0.745, *p* = 0.399, ${\eta }_{p}^{2}$= 0.038) nor the rotation direction of dominant image (*F* (1, 19) = 1.102, *p* = 0.307, ${\eta }_{p}^{2}$= 0.055) was significant. The main effect of the tactile stimulus regarding the dominant image was significant (*F* (2, 38) = 7.411, *p* < 0.005, ${\eta }_{p}^{2}$= 0.281). There was a significant interaction among the three factors (*F* (2, 38) = 5.530, *p* < 0.01, ${\eta }_{p}^{2}$ = 0.218).

For the simple two-way interactions and simple simple-main effects analyses, the overall *α* level of 0.05 was preserved by Bonferroni correction. There was a significant simple two-way interaction between dominant images and rotation direction of visual image under the congruent (*F* (1, 19) = 4.603, *p* < 0.05, ${\eta }_{p}^{2}$ = 0.195) and incongruent (*F* (1, 19) = 5.899, *p* < 0.05, ${\eta }_{p}^{2}$ = 0.237) conditions. No other interaction was significant. There were significant simple simple-main effects of the rotation direction of image for the congruent artificial turf condition and the incongruent bathmat condition (both *p* < 0.05).

We conducted post-hoc tests for tactile stimulus regarding the dominant image factor. For these tests, the overall *α* level of 0.05 was preserved by Bonferroni correction. The total dominant time was significantly longer under the congruent condition than under the no-stimulus and incongruent conditions (both *p* < 0.05). There was no significant difference between the incongruent condition and the no-stimulus condition (*p* = 0.083).

### Discussion

In this experiment, we investigated whether the results of Experiment 1 were due to response bias caused by the semantic information of the stimuli used. For this purpose, we rotated the two visual stimuli in, opposing directions and instructed the participants to report which of the rotated stimuli was dominant in percept. This prevented them from relying on the semantic information of the stimuli. The results were similar to those of Experiment 1, in that the tactile stimulus congruent to either of the two visual stimuli prolonged the dominant time for that visual stimulus. The incongruent tactile stimulus slightly shortened the dominant time, but there was no significant difference between the incongruent condition and the no-stimulus condition.

In Experiment 3, we generalized the findings of the last two experiments using stimuli different from the artificial turf and bathmat.

## Experiment 3

### Methods

#### Participants

Twenty-two students and one postdoctoral researcher from Kyushu University (12 males, 11 females, mean age 19.84 in a range of 18 to 31) participated for payment. They did not participate in either of the previous two experiments. They were unaware of the purpose of this experiment. Prior to the study, procedures were explained to the participants and their informed consent was received. All participants had normal or corrected-to-normal vision and all but one were right-handed.

#### Stimuli and apparatus

**Fig. 6:**
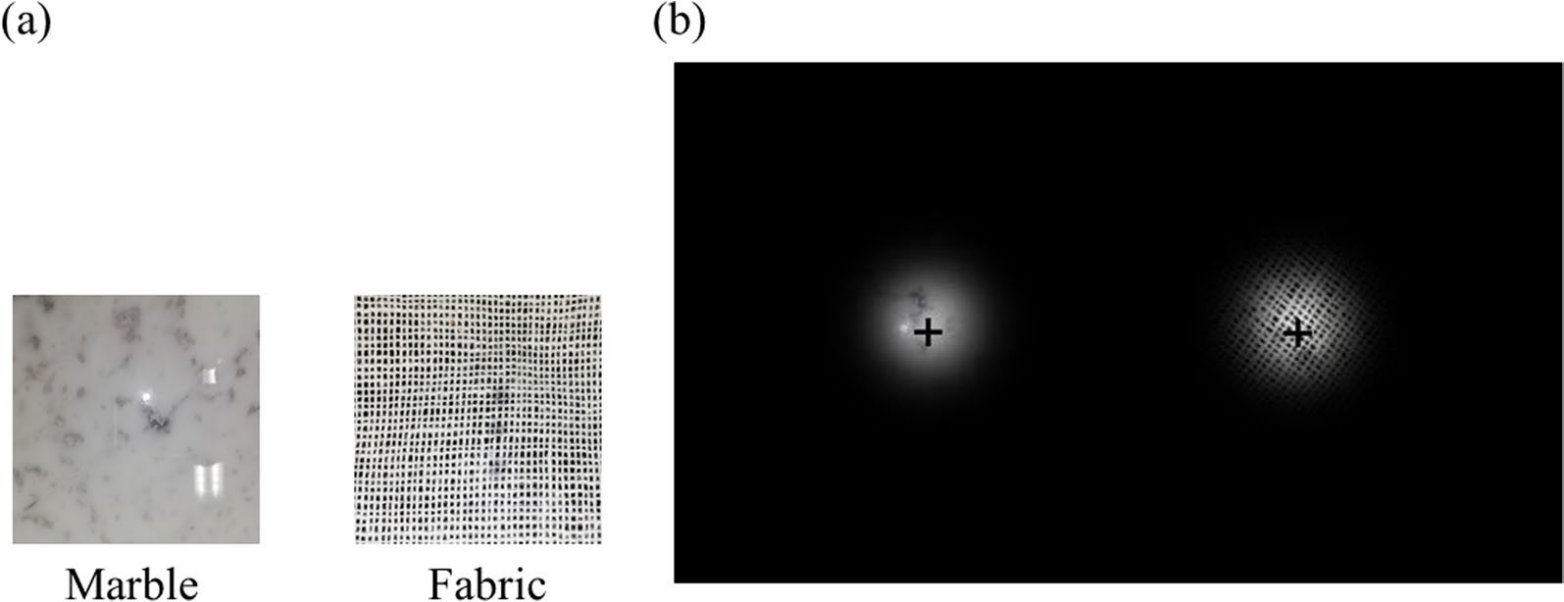
**a** Sample photo of tactile stimuli. **b** Image of presented visual stimuli on the CRT display

Tactile stimuli were a slice of natural marble stone and a sheet of woven fabric (Fig. 6a), both approximately 20 × 20 cm in size. The slice of marble was 1-cm thick, and its surface was flat and polished smoothly. The woven fabric was made of cotton with roughly 0.1-cm mesh, and its surface was uneven and rough. As shown in Fig. 6a, which are colored images, the surface of both stimuli was almost black and white, unlike the stimuli used in Experiments 1 and 2. With these stimuli, we eliminated possible effects of surface color on binocular rivalry. Visual stimuli were circularly cut-out images (6.4° in diameter) of the surfaces of the marble and the fabric, with a mean luminance of 16.39 and 16.26 cd/m^2^, respectively. In order to blur their edge, each image was windowed by 2D-Gaussian (*σ* = 20 pixel). The visual stimuli were presented side-by-side on a CRT display with a black background (Fig. 6b), and were rotated continuously during a 60 s observation period, one CW and the other CCW, at a rate of one degree per frame (frame rate of 75 Hz). The procedures and apparatus were identical to those in Experiment 2.

### Results

**Fig. 7:**
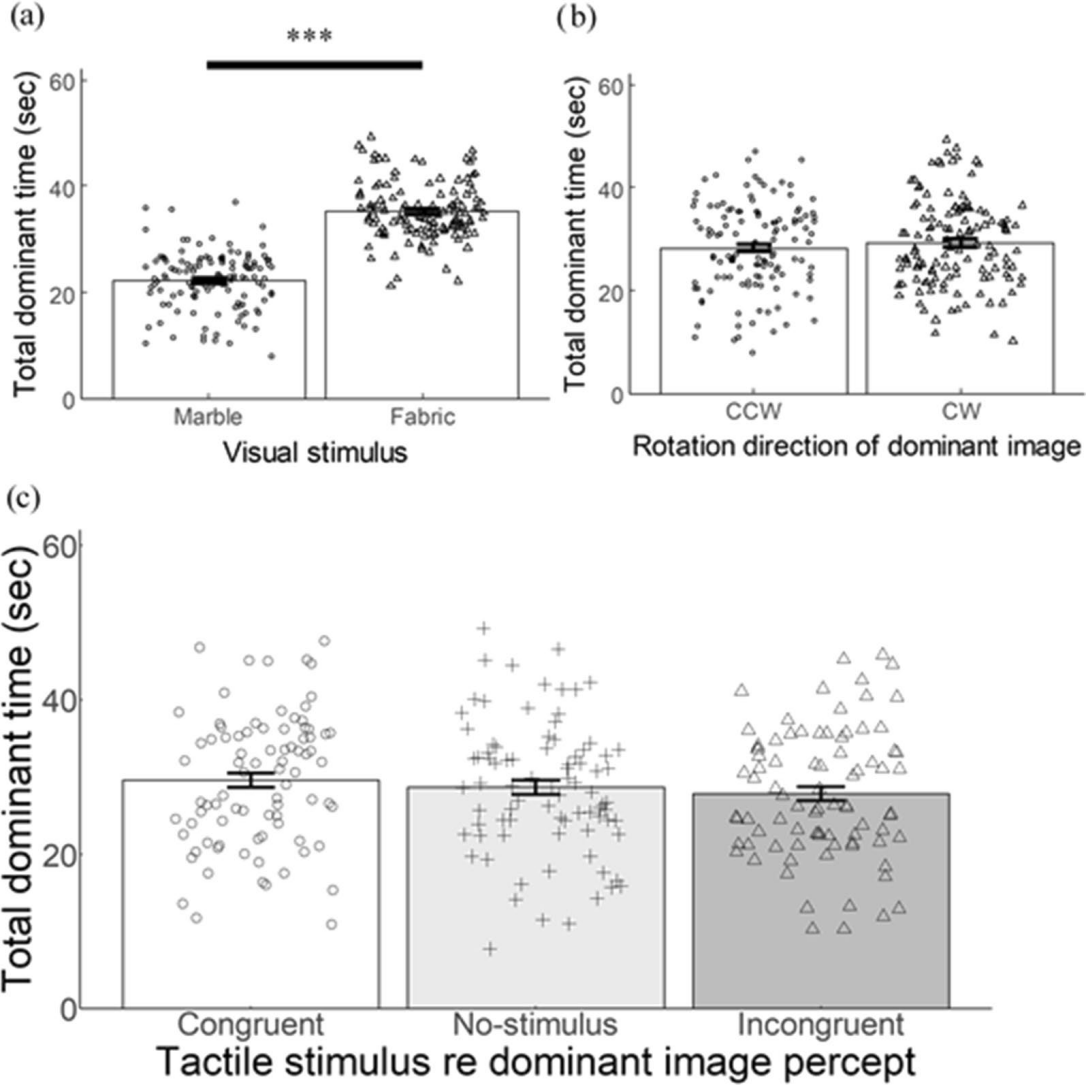
**a** The mean total dominant time for the images of marble and fabric. **b** The mean total dominant time for the rotation direction of dominant images for counter-clockwise (CCW) and clockwise (CW). **c** The mean total dominant time for the tactile stimulus regarding the dominant image. Error bars represent ± 1 SEM. Each dot represents individual data in each condition. *** refer to *p* < 0.001

Data from two participants were removed because they did not report alternating percepts during binocular viewing. The mean total dominant time of twenty-one participants for the dominant image, for the rotation direction of the dominant image, and for the tactile stimulus regarding the dominant image are shown in Fig. 7a–c, respectively. The three-way repeated-measures analysis of variance was performed on the total dominant time with three factors, dominant image (marble or fabric), tactile stimulus regarding the dominant image (congruent, no-stimulus, or incongruent), and rotation direction of the dominant image (CCW or CW). There were significant main effects of the dominant image (*F* (1, 20) = 42.352, *p* < 0.001, ${\eta }_{p}^{2}$= 0.679) and the tactile stimulus (*F* (2, 40) = 4.487, *p* < 0.05, ${\eta }_{p}^{2}$= 0.183). The main effect of the rotation direction of dominant image was not significant (*F* (1, 20) = 4.228, *p* = 0.053, ${\eta }_{p}^{2}$= 0.175). No interaction of the three factors was significant.

We conducted post-hoc tests for the tactile stimulus regarding the dominant image factor. For these tests, the overall *α* level of 0.05 was preserved by Bonferroni correction. There was no significant difference between any pair of the three tactile conditions.

### Discussion

In this experiment, we generalized the findings of the last two experiments using new stimuli, marble and fabric. The significant main effect of the tactile stimulus regarding the dominant image confirmed the results of the previous two experiments, i.e., the tactile stimuli affected binocular rivalry. We found no significant differences among the three tactile stimulus conditions. We have no definitive answer for this, but one possibility is the lack of clear difference in roughness between the visual stimuli, which was unmatched with the clear difference between the corresponding tactile stimuli. As shown in Fig. 6, the visual images of the marble and fabric did not give a clear impression of difference in roughness between them, whereas the tactile roughness was clearly different between the polished surface of the marble and the uneven mesh of the fabric.

As shown in Fig. 7a, the total dominant time of the fabric was significantly longer than that of the marble. The texture of the fabric looked clear and salient compared with the surface of marble. This difference in texture may have led to the difference in the total dominant time with visual stimuli. The important point, however, is that there were effects of visuo-tactile interaction regardless of whether the dominant percept was of the marble or of the fabric.

## General discussion

We investigated whether the visuo-tactile interaction on binocular rivalry is caused by roughness using naturalistic stimuli. In Experiment 1, we used artificial turf (rough) and a bathmat (smooth), and the congruent tactile stimulus prolonged, whereas the incongruent stimulus shortened the total dominant time of visual stimulus. In Experiment 2, we rotated the two visual stimuli rotated in opposite directions and asked the participants to report the dominant image of the rotated stimulus in either direction in order to eliminate possible effects of semantic information of the stimuli. The results were similar to those of previous experiment, suggesting that roughness information of the tactile stimuli affected binocular rivalry, with no influence of response bias. In Experiment 3, we used two new stimuli, smooth marble and rough fabric, to assess whether the results of the previous two experiments are generalizable to stimuli other than artificial turf and bathmat. There was a significant main effect of the tactile stimulus condition. This suggested that the effects of tactile information on binocular rivalry are not limited to particular stimuli. Altogether, these three experiments demonstrated that the visuo-tactile interaction on binocular rivalry was caused by roughness.

One may argue that the present finding of longer dominant time of the congruent condition than that of the no-stimulus condition was due to the absence of hand motion under the no-stimulus condition, as in previous studies (e.g., Lunghi et al., 2010). We consider this unlikely for the following reason. If hand motion affects binocular rivalry, the results of congruent and incongruent conditions may be similar (the total dominant time of two conditions was longer/shorter than that of the no-stimulus condition). In the present three experiments, the total dominant time of congruent and incongruent conditions was respectively longer or shorter than that of the no-stimulus condition. This supports hand motion not affecting our results.

The neural activity involved in binocular rivalry with naturalistic objects was confirmed in the ventral visual cortex (Sheinberg & Logothetis, 1997; Tong et al., 1998). Sheinberg and Logothetis (1997) used monkeys to examine binocular rivalry and found that the response of cells in the inferior temporal cortex through the ventral visual pathway was altered by perceptual alternations. Tong et al. (1998) reported that the dominant visual percepts of the image of human face and house activate the human fusiform face area and parahippocampal area of the ventral visual cortex, respectively, during binocular rivalry. The visuo-tactile interaction of material properties may be involved in ventral visual cortex (Komatsu & Goda, 2018). It is thus plausible that tactile information of material represented in the ventral visual cortex regulates the alternating visual percepts under binocular rivalry.

Our study does not exclude the possibility that the interaction of primitive features, such as spatial frequencies between visual and tactile stimuli, influences binocular rivalry. Lunghi et al. (2010) demonstrated that the influence of tactile information on alternating visual percepts during binocular rivalry was tuned for spatial frequency in that the visuo-tactile interaction was greatest when visual and tactile gratings have the same spatial frequency. They argued that vision and touch interact at early stages of visual processing, probably V1, which has a narrow spatial frequency selectivity.

The observed effects of tactile stimulation on binocular rivalry may be due to the interaction of roughness between visual and tactile stimuli, rather than the interaction of primitive features between them. As for the simplified stimuli, such as visual and tactile gratings, Lunghi and Morrone (2013) suggested that the spatial proximity between visual and tactile stimuli is necessary for visuo-tactile interaction to occur. They reported that when the tactile grating stimulus was positioned 30 cm away (on the horizontal axis) from the visual grating stimulus, the effects of tactile stimulation on binocular rivalry were absent. In the present study, the visual stimuli were not spatially aligned with the tactile stimuli. However, we did not experimentally manipulate the location between visual and tactile stimuli and did not investigate whether their location affects visuo-tactile interaction of roughness. In future studies, we need to examine whether the spatial location of visual and tactile stimuli affects visuo-tactile interaction of roughness on binocular rivalry.

## Data Availability

The datasets generated and/or analyzed during the current study are available from the corresponding author upon reasonable request.
